# Dobutamine administration: a proposal for a standardized approach

**DOI:** 10.1186/s40635-025-00804-1

**Published:** 2025-08-31

**Authors:** Lorenzo Calabró, Filippo Annoni, Fabio Silvio Taccone

**Affiliations:** Department of Intensive Care, Hôpital Universitaire de Bruxelles (HUB), Université Libre de Bruxelles, 1070 Brussels, Belgium

**Keywords:** Dobutamine, Cardiac output, Hemodynamic monitoring, Tissue perfusion, Organ dysfunction, Shock

## Abstract

Dobutamine is the most commonly used inotropic agent in critically ill patients with impaired cardiac contractility. However, its benefit–risk profile remains debated, and clear, structured guidance for its use is lacking. This hypothesis proposes a pragmatic framework for dobutamine administration to promote rational and consistent clinical and experimental practice. The aim is to propose a rational and reproducible use of inotropic therapy with dobutamine in both clinical and experimental settings in cases of shock with low cardiac output, particularly cardiogenic shock, septic shock with septic cardiomyopathy, and low cardiac output syndrome after cardiac surgery (LCOS). Dobutamine should be prescribed only in the presence of acute circulatory failure with signs of peripheral hypoperfusion and impaired cardiac contractility. A low cardiac index (CI) alone does not mandate inotrope initiation. Echocardiography is essential for initial assessment but should be complemented by continuous cardiac output monitoring for evaluating dose–response. The recommended starting dose is 2.5 μg/kg*min, with stepwise titration based on CI and perfusion markers reassessed every 20 min. A significant CI increase and resolution of hypoperfusion should guide further escalation. Persistent hypoperfusion despite CI improvement may indicate inadequate response and justify cautious dose increases, while continued hypoperfusion with further CI rise suggests a flow-independent deficit, discouraging further titration. Dobutamine should be used with clear indications, guided by a standardized approach integrating continuous hemodynamic and perfusion monitoring. This strategy may help optimize therapeutic benefit while minimizing unnecessary exposure and adverse effects.

## Introduction

Dobutamine is the most used inotropic agent worldwide in patients requiring short-term support due to impaired cardiac contractility, regardless of its etiology [1]. Common indications for dobutamine in critically ill patients are cardiogenic shock [2], sepsis-induced myocardial dysfunction with hypoperfusion [3] and LCOS [4]. Several studies have linked dobutamine administration to an increased risk of arrhythmias and mortality, raising concerns regarding its overall benefit–risk profile [5–8]. However, it is more plausible that the requirement for inotropic support was a marker of underlying disease severity, rather than exclusively representing an independent causal factor contributing to adverse outcomes.

Like any vasoactive drug, dobutamine should be prescribed to achieve a specific therapeutic goal. In particular, it should not be administered solely to improve left ventricular ejection fraction or stroke volume, but to address systemic hypoperfusion due to reduced cardiac contractility. As such, drug effects depend on proper patient selection and dose personalization. Unfortunately, practices related to dobutamine initiation and titration vary widely and are inconsistently reported in clinical studies, making outcomes difficult to compare [1]. This viewpoint aims, therefore, to offer a structured and practical approach to dobutamine administration, promoting consistent and rational use.

## First: report the indication

Dobutamine is indicated in acute circulatory failure with persistent hypoperfusion secondary to reduced ventricular systolic function and low cardiac index (CI). As such, a low CI alone does not necessarily require inotropic support if tissue perfusion is adequate; thus, in the presence of signs of tissue hypoxia, suggesting an imbalance between oxygen transport (DO_2_) and oxygen consumption (VO_2_), interventions may become necessary. Also, echocardiography is essential for the initial assessment of cardiac contractility [9], but cannot provide continuous monitoring. Notably, CI may be overestimated by continuous monitoring systems when it is extremely low [10].

In septic patients with apparently preserved ejection fraction or stroke volume but ongoing hypoperfusion, systemic oxygen transport may still be inadequate in regard to tissue requirements. In the absence of hypoxemia, severe anemia and/or hypovolemia, a “dobutamine challenge” has been proposed as a dynamic test to unmask DO_2_/VO_2_ dependency in these critically ill patients [11]. This is characterized by a dose-dependent increase in CI and DO_2_, accompanied by a significant rise in VO_2_; a key indicator of true DO₂/VO₂ dependency could be identified by a minimal or non-proportional elevation in central or mixed venous oxygen saturation (ScvO_2_–SvO₂), improved signs of hypoperfusion, including reduced lactate levels, which would reflect increased cellular oxygen extraction. In this case, the recommended test dose remains within the range of 5–10 µg/kg*min. Notably, the inodilatory properties of dobutamine may exert a beneficial effect on the microcirculation independently of CI increase—a therapeutic strategy currently under investigation in the ANDROMEDA-SHOCK 2 trial [12].

## Second: implement hemodynamic monitoring

Before starting dobutamine, we consider continuous cardiac output monitoring to be essential to assess dose–response relationships and allow precise monitoring. Calibrated transpulmonary thermodilution and uncalibrated pulse contour wave analysis (PCWA) are suitable options. However, both methods have limitations. Transpulmonary thermodilution is less reliable in patients with severe pulmonary hypertension, right ventricular dysfunction, arrhythmias, low tidal volumes in mechanically ventilated patients, and/or valvular disease [10]. PCWA may be inaccurate in severe peripheral vascular disease or in case of suboptimal arterial signal [9]. In patients receiving mechanical circulatory support (e.g., ECMO, IABP, LVAD), these tools may also fail to provide accurate data. In such cases, a pulmonary artery catheter (PAC) may offer more reliable measurements, although some inaccuracy could still be present. An initial echocardiographic exam should guide the choice of monitoring system based on the patient's hemodynamic profile and comorbidities; however, its role as a sole monitoring tool is limited by the time-consuming nature of repeated evaluations and its reliance on operator skill and experience [9].

Cardiac index should be reassessed 20 min after any change in dobutamine dosage to evaluate its therapeutic effect. However, the optimal threshold for defining a clinically meaningful increase in CI, e.g., identifying CI responders, remains uncertain. In this setting, a 15% increase in CI, which is frequently employed as the minimum threshold to define a significant hemodynamic response in fluid responsiveness studies [13], may be extrapolated to assess inotropic responsiveness. Although this threshold remains largely arbitrary, it represents a pragmatic compromise to account for the inherent variability of CI measurements, thereby reducing the risk of false-positive responses with lower thresholds and minimizing underestimation of clinically relevant changes by using higher thresholds. Concurrent trends in pulmonary artery wedge pressure (PAWP) may offer additional insights into ventricular pre-load reserve and compliance; however, PAWP should not serve as the primary therapeutic target [14].

## Third: standardized dose prescription

The lowest effective dobutamine dose reported in pharmacokinetic studies is 2.5 μg/kg min [15], which appears sufficient to achieve meaningful hemodynamic changes without significantly increasing myocardial oxygen consumption. A reasonable strategy is therefore to start at 2.5 μg/kg min and titrate the dose by 2.5 μg/kg min increments (e.g., 2.5, 5, 7.5, 10), reassessing after each adjustment until hypoperfusion resolves [16]; the goal remains to use the lowest effective dose. After each change, CI and perfusion markers should be reevaluated after 20 min. The maximum recommended dose is approximately 20 μg/kg min [2]; beyond this, receptor saturation may limit further contractility improvement, while carrying a significant increased risk of side effects, such as tachycardia and hypotension [17]. If CI increases mainly due to heart rate, with minimal change in stroke volume, this may indicate exhausted inotropic reserve, where further dose escalation increases the risk of arrhythmias and myocardial oxygen demand without benefit.

## Fourth: always monitor tissue perfusion

Dobutamine therapy should be guided by signs of peripheral hypoperfusion, secondary to reduced ventricular systolic function (and low CI) or insufficient DO2 (“dobutamine challenge”) [18]. Common clinical indicators of peripheral hypoperfusion include: (1) mottled skin, assessed via mottling score, though unreliable in chronic peripheral vasculopathy [19]; (2) capillary refill time (CRT) > 3 s, which is also affected by peripheral vascular disease [20]; (3) new-onset encephalopathy, typically presenting as lethargy or obnubilation, possibly related to hypoperfusion, but often being multifactorial; (4) persistent oliguria, which may result from hypoperfusion but can also arise from obstructive or intrinsic renal causes; (5) signs of bowel ischemia, however infrequent and difficult to monitor and diagnose, particularly in the absence of surgical complications (e.g., peritonitis, malignancy).

Together with clinical ones, biological indicators of peripheral hypoperfusion are: (1) low ScvO₂ or SvO₂ (< 65%), indicating increased oxygen extraction. When anemia, hypoxemia, or high VO_2_ are excluded, such values typically reflect inadequate CI and low DO_2_. Importantly, low ScvO_2_–SvO_2_ may be observed in patients with compensated chronic heart disease [21], and should not be used alone to initiate dobutamine; (2) elevated veno-arterial pCO₂ gap (> 6 mmHg), suggesting impaired CO₂ clearance due to low CI, though it may also reflect increased peripheral metabolism [22]; (3) hyperlactatemia (> 2 mmol/L), indicative of anaerobic metabolism from insufficient DO_2_, though other causes such as hepatic dysfunction, mitochondrial impairment, adrenergic stimulation, or drug toxicity must be considered [23].

Importantly, although each of these signs or biomarkers may serve as a sensitive indicator of peripheral hypoperfusion, none is sufficiently specific on its own to warrant clinical intervention. As such, the simultaneous presence of at least two pathological indicators offers a more accurate and reliable basis for diagnosing peripheral hypoperfusion (Table 1), suggesting the need for a multimodal assessment of tissue perfusion. Moreover, additional quantitative tools for assessing tissue perfusion, such as sublingual video-microscopy, tissue near-infrared spectroscopy (NIRS), and laser Doppler flowmetry, could also be considered. However, their clinical utility remains undefined due to limited availability, insufficient validation in critically ill populations, and the need for specialized skills and expertise, which constrain their widespread applicability.

**Table 1:** Markers of tissue hypoperfusion and their limitations

Markers of tissue hypoperfusion	Limitations
Peripheral mottling	Unreliable if chronic peripheral vasculopathy
Capillary refill time > 3 s	Unreliable if chronic peripheral vasculopathy
Encephalopathy	Not specific (other etiologies are possible)
Persistent oliguria	Secondary to kidney injury of renal or obstructive origin Previous renal disease
Venous oxygen saturation < 65%	Hypoxemia and severe anemia May occur in the absence of tissue hypoperfusion
Δv-a pCO_2_ > 6 mmHg	High metabolic activity May occur in the absence of tissue hypoperfusion
Lactate levels > 2 mmol/L	Inadequate hepatic clearance (e.g., liver disease) Mitochondrial dysfunction Drug toxicity May occur in the absence of tissue hypoperfusion

Δv-a pCO_2_: arterio-venous difference carbon dioxide partial pressure

**Fig. 1:**
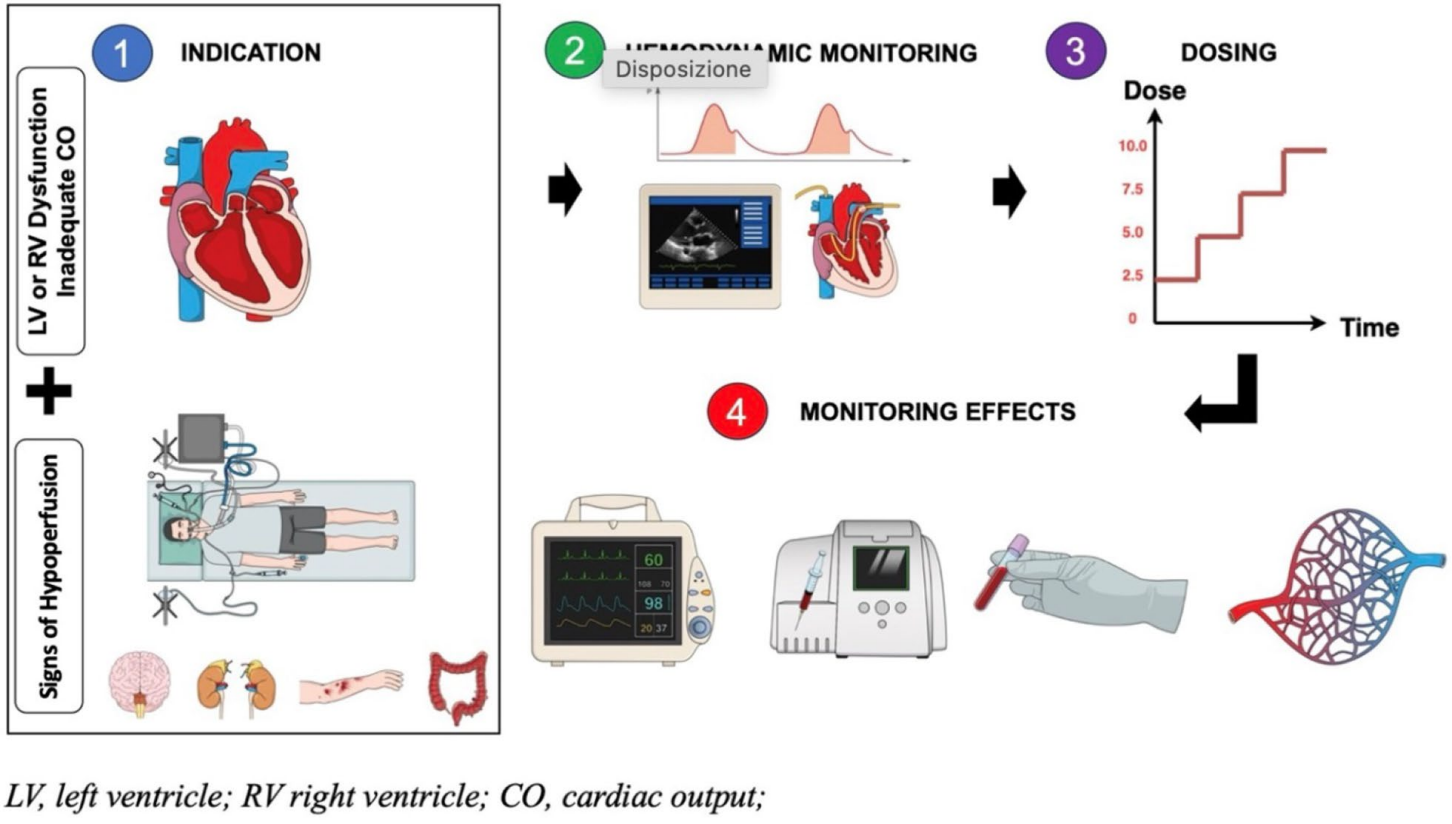
A structured approach for dobutamine administration

Any change in dobutamine dosing should be followed by reassessment after 20 min, evaluating both CI and tissue perfusion (Fig. 1). Improvement in both suggests therapeutic efficacy. A reduction or resolution of hypoperfusion in the absence of an increase in CI with dose escalation may indicate a microcirculatory effect of dobutamine or the presence of a confounding factor during the test, which should be investigated. Persistent signs of hypoperfusion despite an initial increase in CI may indicate an inadequate hemodynamic response, warranting cautious escalation of dobutamine dosage to evaluate whether higher doses yield improved tissue perfusion. In this context, echocardiography may be again very useful to reassess contractility and provide indexes of pre-load dependency. However, if hypoperfusion persists in absence of or despite further increases in CI, this may suggest a flow-independent mechanism of impaired perfusion, in which case additional dose escalation is unlikely to provide clinical benefit and therapy discontinuation should be considered. Likewise, if dose escalation is accompanied by worsening hypotension without improvement in hypoperfusion, the dose should be reduced or therapy discontinued.

In the event of an insufficient response to, or intolerance of, dobutamine in scenarios such as cardiogenic shock or LCOS, escalation to mechanical circulatory support should be considered [24]. Alternative inotropes to dobutamine, such as milrinone and levosimendan, have not demonstrated significant improvements in primary outcomes in randomized trials [16, 25]; however, their use in place of dobutamine may have a role in selected cases and warrants a case-by-case discussion.

## Conclusions

Dobutamine should be reserved for acute circulatory failure with two or more signs of peripheral hypoperfusion and impaired contractility. Continuous cardiac output monitoring is mandatory in patients receiving dobutamine. Clinicians should start at the lowest possible dose, reassess CI and perfusion after 20 min, look for a significant increase in CI primarily from stroke volume and resolution of hypoperfusion. Stepwise dose titration should prompt for repeated hemodynamic assessment after each adjustment. The same approach should be applied during drug weaning. The maximum recommended dose is 20 μg/kg·min.

## Data Availability

Data available within the article or its supplementary materials.

## Abbreviations

CI: Cardiac index
PCWA: Pulse contour wave analysis
PAC: Pulmonary artery catheter
PAWP: Pulmonary artery wedge pressure
ScvO_2_: Central venous oxygen saturation
SvO_2_: Mixed venous oxygen saturation
NIRS: Near-infrared spectroscopy
